# Supplementary figures and images for: Soma to neuron communication links stress adaptation to stress avoidance behavior
Source: bioRxiv. 2025 Sep 5:2025.05.07.652728. Originally published 2025 May 7. Preprint. [Version 2] doi: 10.1101/2025.05.07.652728 (PMC12248090; doi:10.1101/2025.05.07.652728)

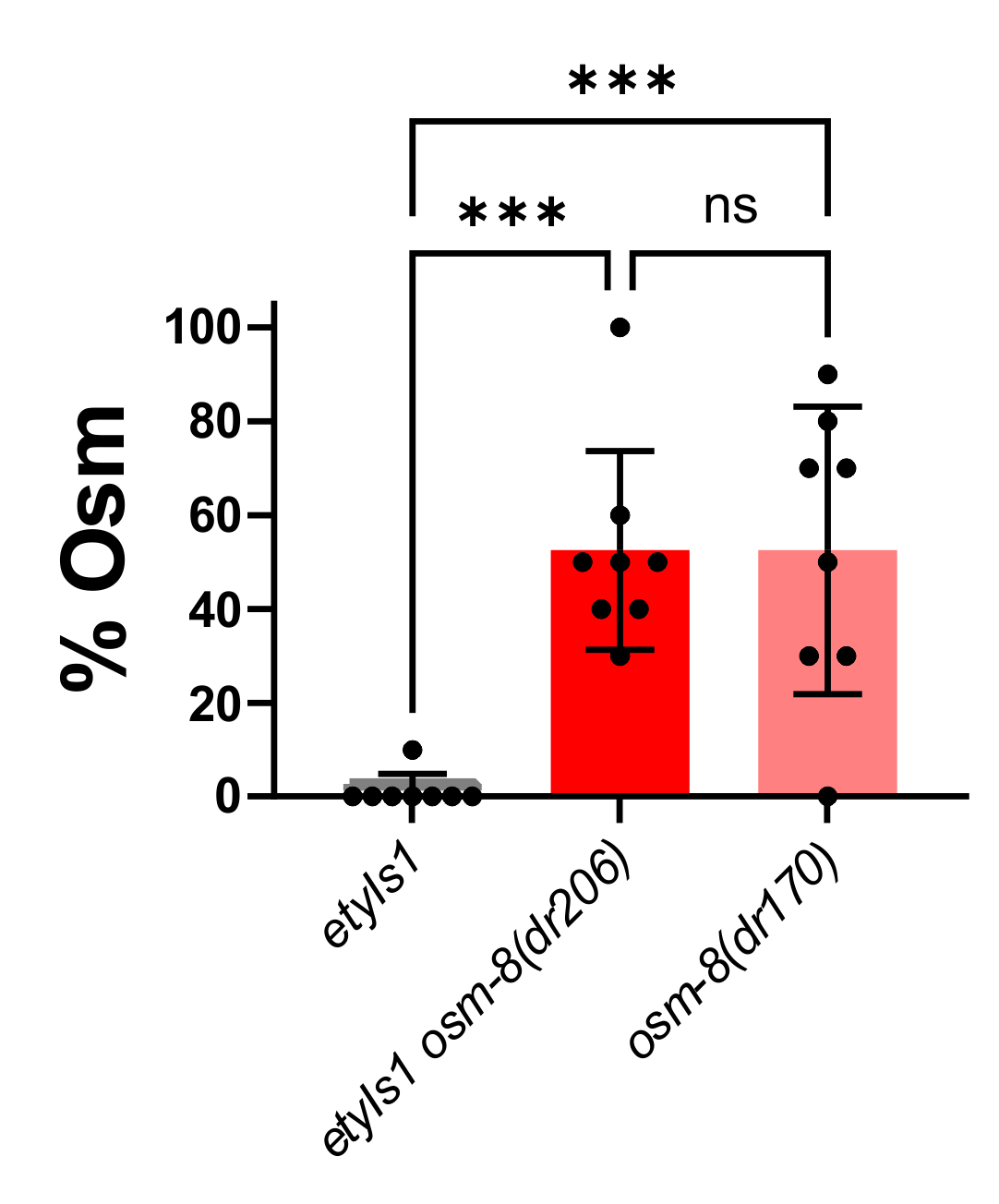

Supplement: Supplement 1 — Figure S1 – Osmotic avoidance behavior in wild type and osm-8 mutant animals with or without the etYIs1 GCaMP6s transgene. N=8 replicates per genotype (10 animals per replicate, N=80 per genotype). ****- p<0.0001, ‘ns’-not significant, One-way ANOVA with Tukey post hoc test. Individual data points are shown along with the mean ± S.D. [file media-1.tif]

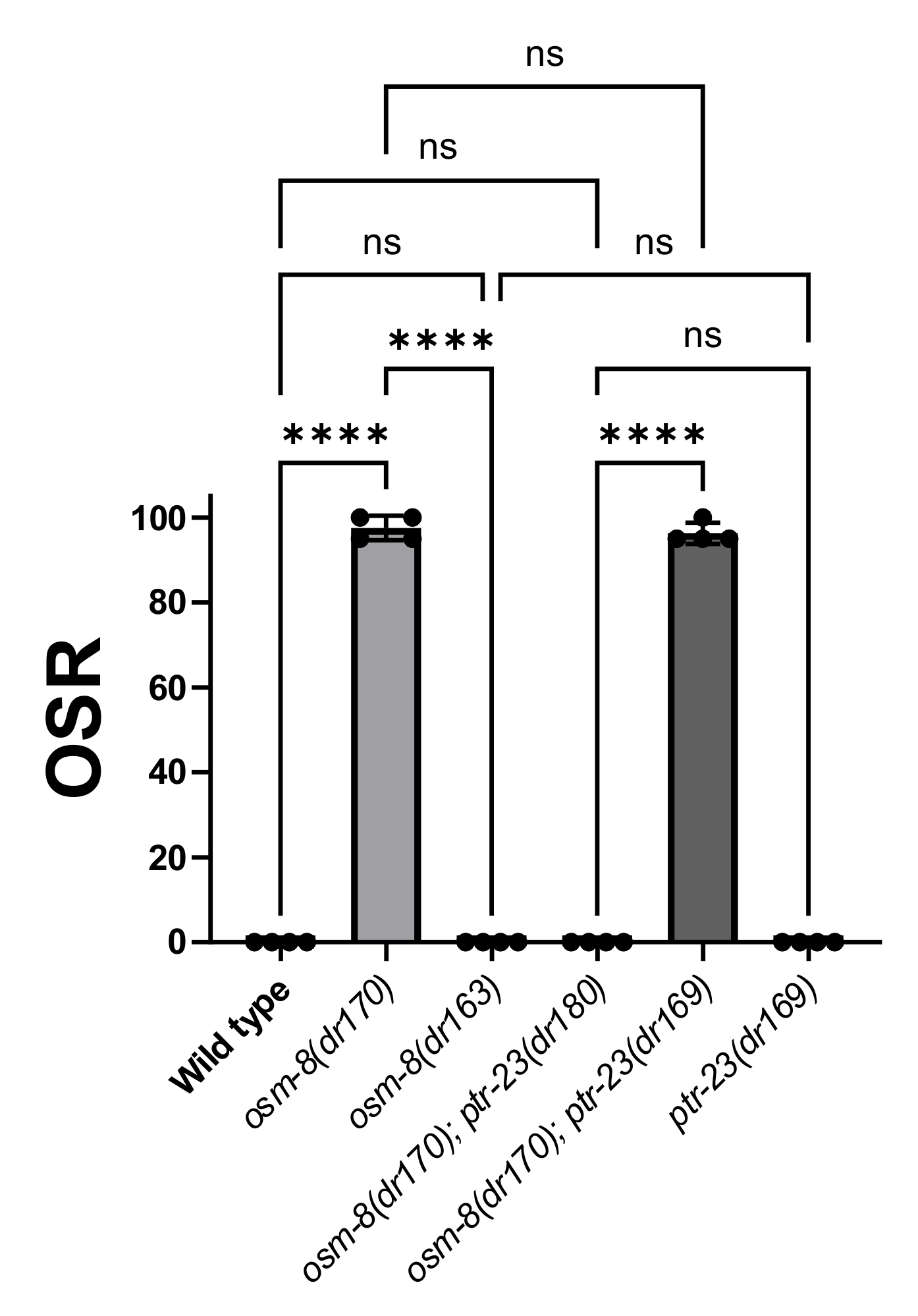

Supplement: Supplement 2 — Figure S2 – Osmotic stress resistance phenotype of tagged osm-8 and ptr-23 alleles. N=4 replicates per genotype (20 animals per replicate, N=80 per genotype). ****-p<0.0001, ‘n.s.’ – not significant, One-way ANOVA with Tukey posthoc testing. [file media-2.tif]

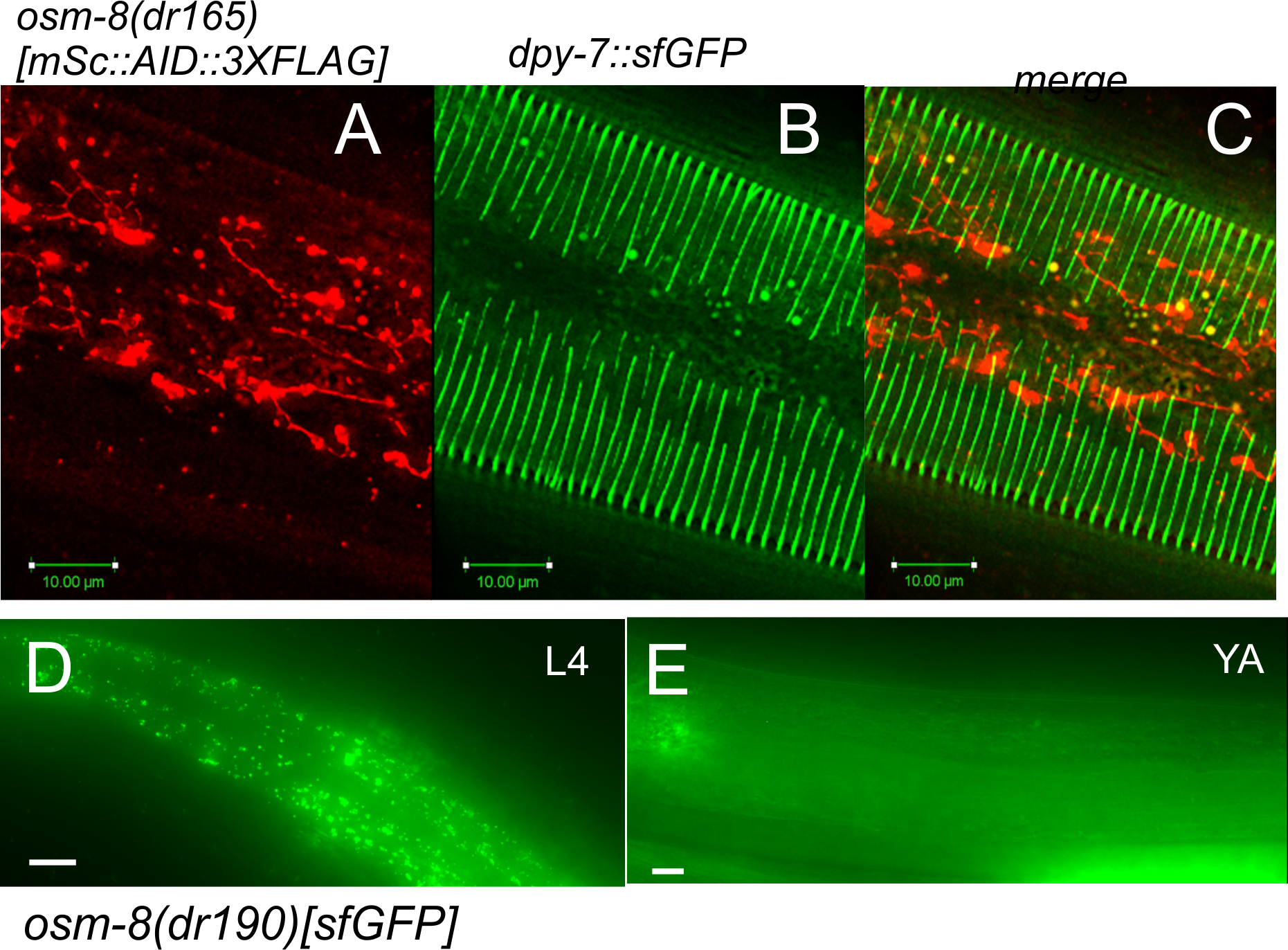

Supplement: Supplement 3 — Figure S3 – Localization of endogenously tagged osm-8 alleles. A) osm-8(dr165) [mScarlet CRISPR allele] B) dpy-7::sfGFP C) merge. Scale bar = 10 microns. D) osm-8(dr190) [sfGFP CRISPR allele] in L4 or E) day 1 adult animals. Scale bar = 10 microns. [file media-3.tif]

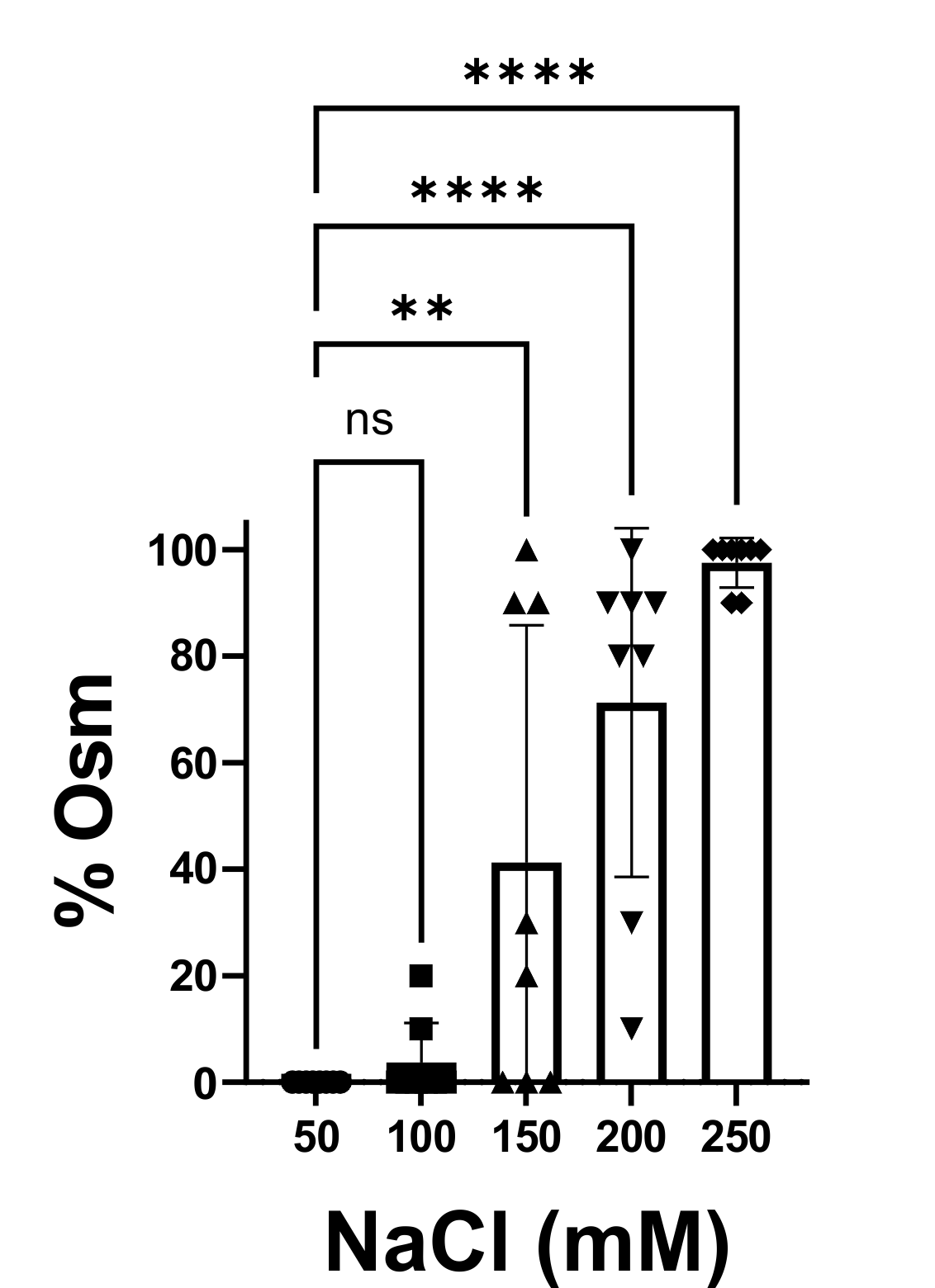

Supplement: Supplement 4 — Figure S4 – Dose response of osmotic adaptation-induced Osm behavior in wild type animals. Adaptation period was 24 hours. N=8 replicates per genotype and per time point (10 animals per replicate, N=80 per genotype for each concentration). ****-p<0.0001, ‘ns’-not significant, One-way ANOVA with Tukey post hoc test. Individual data points are shown along with the mean ± S.D. [file media-4.tif]

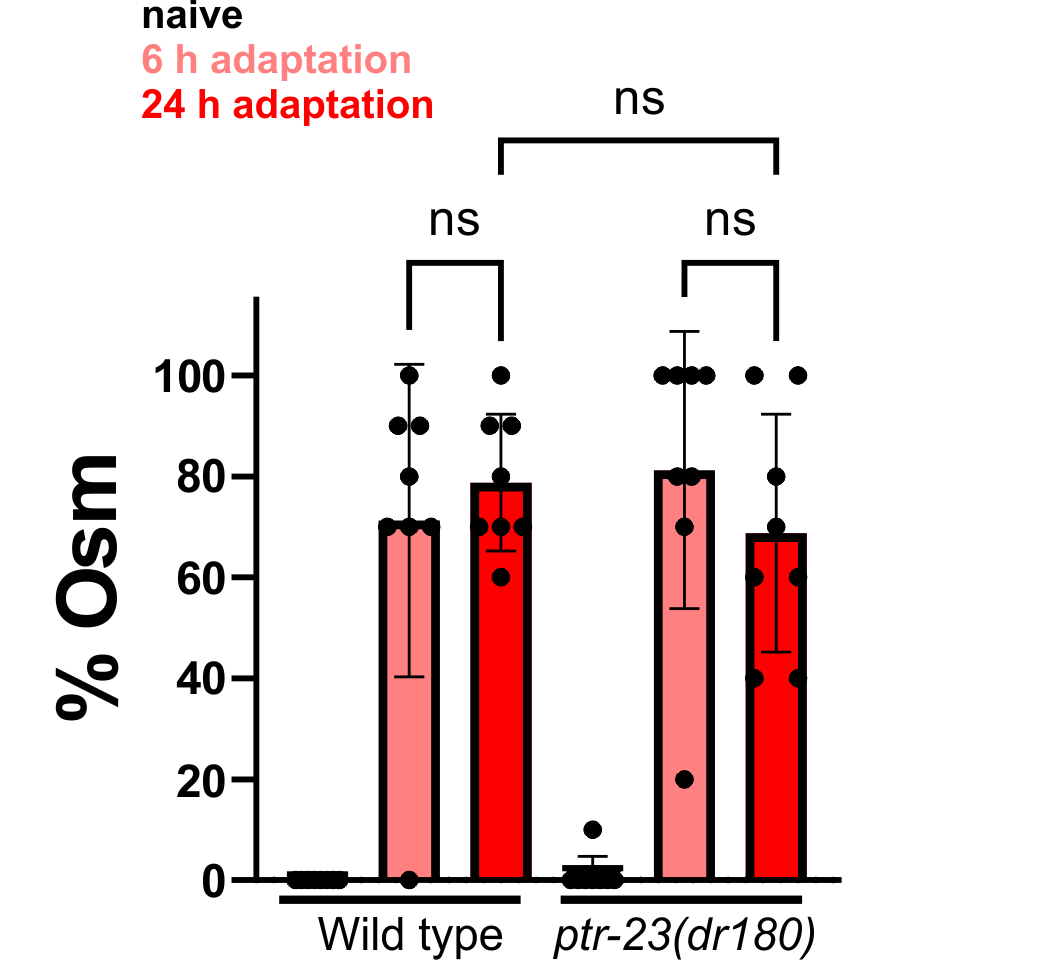

Supplement: Supplement 5 — Figure S5 – Adaptation induced Osm behavior in wild type and ptr-23(dr180) mutants. N=8 replicates per genotype and per time point (10 animals per replicate, N=80 per genotype for each time point). ****-p<0.0001, ‘ns’-not significant, One-way ANOVA with Tukey post hoc test. Individual data points are shown along with the mean ± S.D. [file media-5.tif]

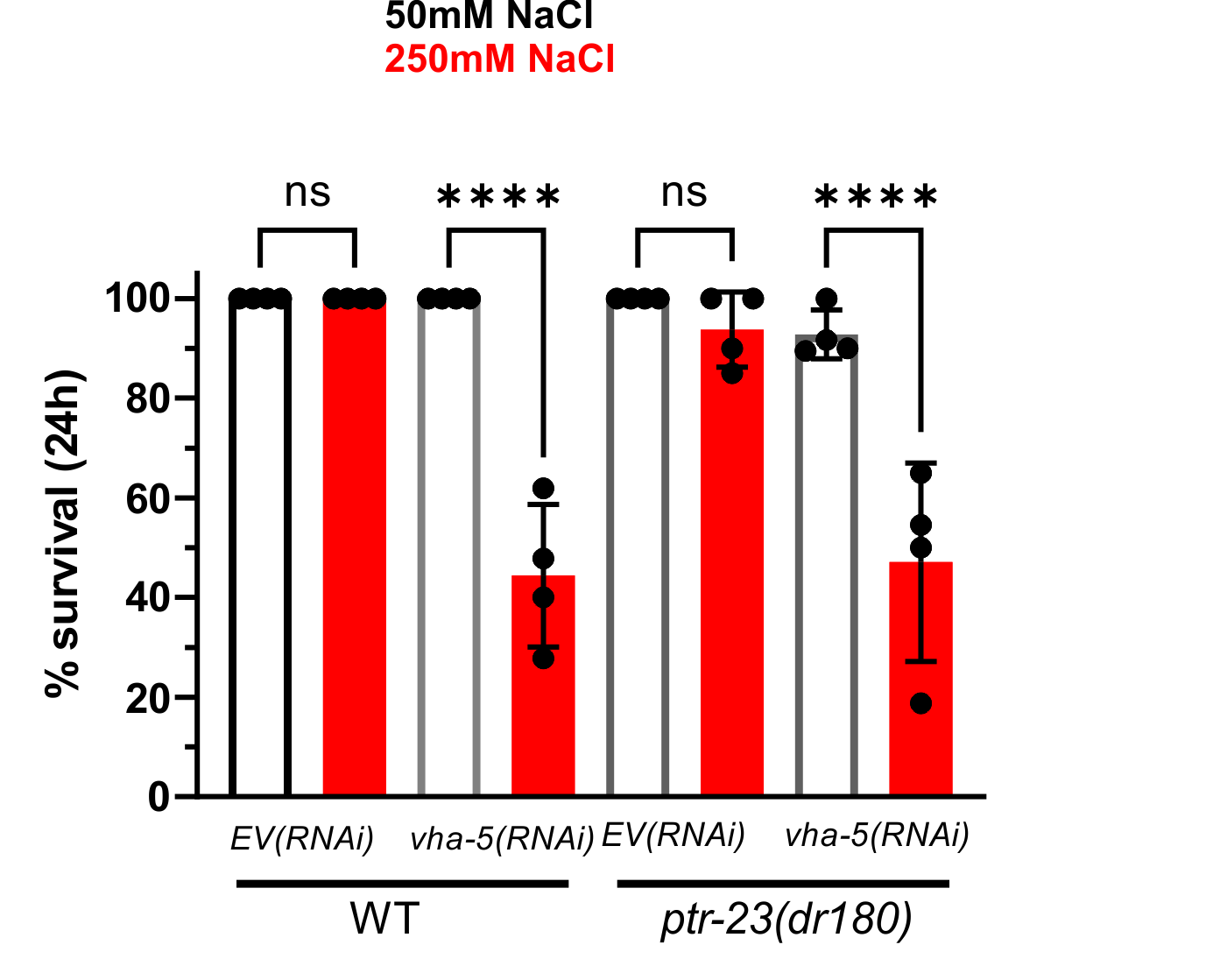

Supplement: Supplement 6 — Figure S6 - Survival of vha-5(RNAi) day 1 adults in either wild type or ptr-23(dr180) after 24 hour exposure to NGM plates with either 50mM NaCl or 250mM NaCl. N=4 replicates per condition (20-25 animals per replicate, 80-100 animals per condition per genotype). ****-p<0.0001, ‘ns’-not significant, One-way ANOVA with Tukey post hoc testing. Individual data points are shown along with the mean ± S.D. [file media-6.tif]

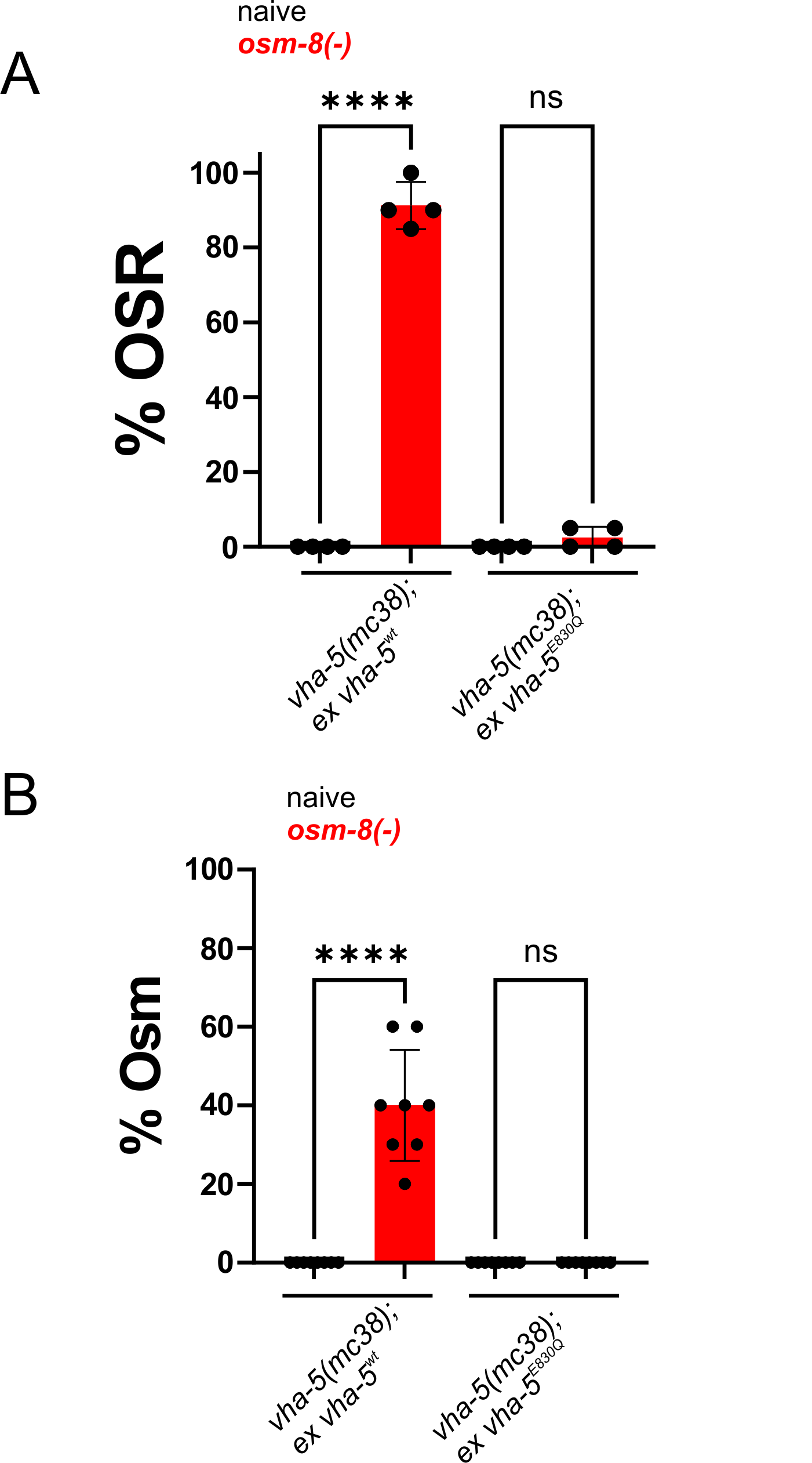

Supplement: Supplement 7 — Figure S7 – Non-canonical V-ATPase independent functions of vha-5 are required for osm-8 phenotypes. A) OSR phenotype of wild type or osm-8 mutants in the vha-5 null mutant mc38 with rescue from either a vha-5(+) wild type or vha-5E830Q mutant transgene. N=4 replicates per genotype (20 animals per replicate, N=80 per genotype). ****- p<0.0001, ‘n.s.’ – not significant, One-way ANOVA with Tukey post hoc testing. B) Osm behavior in wild type or osm-8 mutants in the vha-5 null mutant mc38 with rescue from either a vha-5(+) wild type or vha-5E830Q mutant transgene. N=8 replicates per genotype and per time point (10 animals per replicate, N=80 per genotype for each time point). ****-p<0.0001, ‘ns’-not significant, One-way ANOVA with Tukey post hoc test. Individual data points are shown along with the mean ± S.D. [file media-7.tif]
