# Supplementary material for: Soma to neuron communication links stress adaptation to stress avoidance behavior
Source: bioRxiv. 2025 Sep 5:2025.05.07.652728. Originally published 2025 May 7. Preprint. [Version 2] doi: 10.1101/2025.05.07.652728 (PMC12248090; doi:10.1101/2025.05.07.652728)
Supplement: 8 [file NIHPP2025.05.07.652728v2-supplement-8.pdf]

bioRxiv preprint doi: https://doi.org/10.1101/2025.05.07.652728; this version posted September 5, 2025. The copyright holder for this preprint (which was not certified by peer review) is the author/funder, who has granted bioRxiv a license to display the preprint in perpetuity. It is made available under aCC-BY 4.0 International license.

Video S1. Wild type osmotic avoidance behavior. Ring assay with 4M NaCl. Spot of OP50 *E. coli* in upper right quadrant provides both a motivating stimulus to cross the ring and a ‘sink’ to retain worms that cross the barrier to prevent them from re-entering the ring. Note that wild type animals exhibit chemotaxis towards the OP50 stimulus even when inside the ring, consistent with an odorant-based attractive mechanism.

Video S2. *osm-6(p811)* osmotic avoidance behavior. Ring assay with 4M NaCl. Spot of OP50 *E. coli* in upper right quadrant provides both a motivating stimulus to cross the ring

bioRxiv preprint doi: https://doi.org/10.1101/2025.05.07.652728; this version posted September 5, 2025. The copyright holder for this preprint (which was not certified by peer review) is the author/funder, who has granted bioRxiv a license to display the preprint in perpetuity. It is made available under aCC-BY 4.0 International license.

and a ‘sink’ to retain worms that cross the barrier to prevent them from re-entering the ring. Note that *osm-6* mutants do not appear to chemotaxis towards the OP50 stimulus, consistent with their general defect in both soluble and odorant-based sensory neuron function.

Video S3. *osm-8(dr170)* osmotic avoidance behavior. Ring assay with 4M NaCl. Spot of OP50 *E. coli* in upper right quadrant provides both a motivating stimulus to cross the ring and a ‘sink’ to retain worms that cross the barrier to prevent them from re-entering the ring. Note that unlike *osm-6*, *osm-8* mutants chemotaxis towards the OP50 stimulus, consistent with the hypothesis that *osm-8* mutants do not cause a general defect in chemosensory function.
